# The effect of moxidectin 0,1% vs ivermectin 0,08% on milk production in sheep naturally infected by gastrointestinal nematodes

**DOI:** 10.1186/1746-6148-5-41

**Published:** 2009-11-12

**Authors:** Giuseppe Cringoli, Vincenzo Veneziano, Laura Mezzino, Mariaelena Morgoglione, Saverio Pennacchio, Laura Rinaldi, Vincenzo Salamina

**Affiliations:** 1 University of Naples "Federico II": Department of Pathology and Animal Health, Lab. Parasitology and Parasitic Diseases - CREMOPAR Regione Campania, Via della Veterinaria n.1 80137 Napoli Italy; 2 Fort Dodge Animal Health, Via G. Amendola, 8 Bologna 40121 Italy

## Abstract

**Background:**

Gastrointestinal nematode (GIN) infection is one of the main constraints to sheep production both in temperate and tropical countries. Economic losses caused by GIN are related to decreased production, treatment costs and even animal death. The present paper was aimed at assessing the anthelmintic efficacy (based on faecal egg count reduction) of moxidectin and ivermectin both admistered *per os *at dose rate of 0.2 mg/Kg body weight and the benefit of anthelmintic treatments on milk production in a commercial dairy sheep farms in central Italy whose animals were naturally infected by GIN.

**Results:**

The treatment with moxidectin was highly effective (> 98%) from day 7 until day 75, and effective (90-98%) until day 105. The treatment with ivermectin was highly effective (> 98%) from day 7 until day 14, effective (90-98%) at day 28 and moderately effective (80-89%) on day 45. The milk productions in the treated groups were significantly higher than those of the control group.

**Conclusion:**

In conclusion, the results of the present study demonstrated that moxidectin and ivermectin adminstered *per os *according to the manufacturer's instructions were both effective and safe anthelmintics in sheep. The total milk production was higher in the treated groups than the control group. Overall, animals treated with moxidectin had a milk production 40.8% higher than control group; whereas animals treated with ivermectin had a milk production 32.2% higher than control group.

## Background

Gastrointestinal nematode (GIN) infection (caused by different genera of nematodes, e.g. *Teladorsagia*, *Haemonchus*, *Bunostomum, Cooperia, Nematodirus, Trichostrongylus, Chabertia *and *Oesophagostomum*) is one of the main constraints to livestock production both in temperate and tropical countries. In many cases, GIN parasitism can be attributed to a nutritional disease, because the presence of worms usually induces a decrease in appetite, a decreased digestibility of the food and a diversion of nutrients from production sites towards the repair of tissue-damage caused by the parasites. Therefore, economic losses caused by GIN are related to decreased production, treatment costs and even animal death.

Control of these parasitic infections in ruminants relies almost exclusively on multiple and regular dosing with anthelmintics. Besides the parasitological efficacy of an anthelmintic treatment, it is very important to consider its strategic and economic benefits. Indeed, for producers the two primary aims of anthelmintic treatment strategies are firstly to maintain or improve animal performance and secondly to reduce pasture contamination [1].

The beneficial impact of anthelmintic treatment on milk production have been extensively documented in dairy cows [2-9]; however, very few studies have been conducted on this topic in dairy goats [10] and sheep [1,11-13].

The present field trial was aimed at comparing the anthelmintic efficacy and benefit of treatment with Moxidectin 0,1% (Cydectin™ 0.1%, Fort Dodge Animal Health given *per os *at the dosage of 0.2 mg/kg body weight), with that of Ivermectin 0,08% (Oramec™ 0.08%, Merial Animal Health given *per os *at the dosage of 0.2 mg/kg body weight) - both used at the dose rates recommended by manufacturers - on milk production in a commercial dairy sheep farm in central Italy, utilising animals naturally infected by GIN.

## Results

### Necropsies

The necropsies performed 3 days before the beginning of the trial allowed us to identify the species of GIN in the animals of the study farm. The adult nematodes recovered and identified in sheep were: *Teladorsagia circumcincta *and *Haemonchus contortus *in the abomasums, *Trichostrongylus vitrinus *in the small intestine, and *Oesophagostomum venulosum *in the large intestine.

### Faecal egg counts

The arithmetic mean (AM) EPG on Day 0 was 409.0 for the MOX-group, 441.3 for the IVM-group and 394.8 for the C-group. The percentage reductions in faecal egg counts (FECR) in the MOX-group, compared to the untreated C-group, were 100.0% on day 7; 99.9% on day 14 (95% confidence interval (CI) = 99.5-99.9%); 100% on day 28; 98.9% on day 45 (95% CI = 95.1.0-99.7%); 99.5% on day 75 (95% CI = 97.9-99.9%); and 91.1% on day 105 (95% CI = 86.7-98.2%).

Thus, the treatment with moxidectin was highly effective (> 98%) from day 7 until day 75, and effective (90-98%) until day 105.

The FECR in the IVM-group, compared to the untreated C-group, was 99.8% on day 7 (95% CI = 99.5-99.9%); 99.4% on day 14 (95% CI = 98.4-99.8%); 90.2% on day 28 (95% CI = 93.7-96.1%); 82.7% on day 45 (95% CI = 80.7-87.6%); 71.6% on day 75 (95% CI = 63.2-89.6%); and 58.2% on day 105 (95% CI = 20.3-78.1%).

Thus, the treatment with ivermectin was highly effective (> 98%) from day 7 until day 14, effective (90-98%) on day 28 and moderately effective (80-89%) on day 45.

It should be noted that the study animals were also infected by lungworms (Metastrongylidae) with 60 larvae per gram of faeces (LPG) at day 0. The efficacy of moxidectin against lungworms was 100% from day 14 until day 45 and then ranged from 96.0% to 91.0% until day 185. The efficacy of ivermectin against lungworms was 100% at day 14, 98% at day 28 and then ranged from 92.0% to 88.0% until day 185.

### Coprocultures

Regarding the MOX-group, faecal cultures performed at day 0 revealed the presence of the following genera (average on 5 pools representative of the 30 animals of the MOX-group): *Haemonchus *(20%), *Teladorsagia *(40%), *Trichostrongylus *(31%) and *Oesophagostomum *(9%). From day 7 to day 105 post treatment, only very few GIN larvae were found and thus the genus percentage was not determined. Larvae of *Haemonchus*, *Teladorsagia*, *Trichostrongylus *and *Oesophagostomum *were found in different percentages from day 135 to day 185.

Regarding the IVM-group, faecal cultures performed at day 0 revealed the presence of the following genera (average on 5 pools representative of the 30 animals of the IVM-group): *Haemonchus *(18%), *Teladorsagia *(30%), *Trichostrongylus *(42%) and *Oesophagostomum *(10%). From day 7 to day 105 post-treatment only very few GIN larvae were found and thus the genus percentage was not determined. Larvae of *Haemonchus*, *Teladorsagia*, *Trichostrongylus *and *Oesophagostomum *were found in different percentages from day 135 to day 185.

Regarding the C-group, faecal cultures performed at days 0 revealed the presence of the following genera (average on 5 pools representative of the 30 animals of the C-group): *Haemonchus *(22%), *Teladorsagia *(35%), *Trichostrongylus *(29%) and *Oesophagostomum *(14%). Faecal cultures performed until the end of study confirmed the presence of the same 4 genera in different percentages.

### Milk production

The arithmetic means of fortnightly milk production for the treated groups (MOX-group and IVM-group) and control untreated group (C-group) were assessed. In the first 6 milk sampling dates, both MOX-group and IVM-group showed milk productions significantly higher (p < 0.05) than the milk production of the control group. Overall, the mean milk productions of the treated groups were significantly (p < 0.05) higher than those of the control group as follows: 331.1 ml *versus *235.2 ml (+ 40.8%) for MOX-group, and 310.9 ml *versus *235.2 ml (+ 32.2%) for IVM-group. If considered until day 120, mean milk production was as follows: 439.6 ml *versus *304.7 ml (+ 44.3%) for MOX-group, 414.9 ml *versus *304.7 ml (+ 36.2%) for IVM-group.

## Discussion

The results of the present trial demonstrated that moxidectin and ivermectin adminstered *per os *according to the manufacturer's instructions (dose rate of 0.2 mg/kg b.w.) were both effective and safe anthelmintics in sheep.

As expected, moxidectin showed a longer persistence of anthelmintic action compared to ivermectin; this was probably due to the differences in potency, physicochemical properties and pharmacokinetic behaviour between the two drugs. Indeed, moxidectin is much more lipophilic than ivermectin and is mainly stored in fat [14]. This appears to have an accumulatory effect and results in a long mean residence time for the drug in the body, as it has been widely demonstrated in cows [15], in sheep [16], in goats [17], in horses [18] and in water buffaloes [14].

Regarding the economic efficacy of the treatments, the findings of the present study showed a total milk production higher in the treated groups (MOX-group and IVM-group) than the control group. Specifically, overall, animals treated with moxidectin had a milk production 40.8% higher than control group; those animals treated with ivermectin had a milk production 32.2% higher than control group.

These results are in general agreement with studies in dairy sheep (e.g. [12]) that have used lamb liveweights to provide an indirect measure of milk production, and have reported milk yield to be increased by 15% following anthelmintic treatment with moxidectin towards the end of the gestation. Also, recent field trials conducted in southern Italy showed that strategic prophylactic treatment regimes based on the use of moxidectin milk yield in naturally infected ewes by between 4 and 44% [1,11].

## Conclusion

Effective chemical anthelmintics remain irreplaceable for worm control and their elimination is not practical on animal welfare and economic grounds. Even if the effect of macrocyclic lactones on GIN was demonstrated several years ago, in the era of anthelmintic resistance, it is still important that farmers and veterinarians are concerned with parasitological, strategic, production and economic benefits accruing from prophylactic anthelmintic treatments.

## Methods

The trial was conducted between January and August 2007 on a commercial sheep farm located in the municipality of Bojano, province of Campobasso, central Italy. The flock consisted of approximately 1700 sheep of Gentile di Puglia and cross breeds (Île-de-France × Gentile di Puglia).

### Flock parasitological status

The sheep had naturally acquired mixed parasite infections. The species of GIN in the animals were identified 3 days before the beginning of the trial, by slaughter and necropsy of three sheep randomly selected from animals coprologically positive for GIN. The viscera were processed for sample collection and further worm counts and identification of parasites present in the abomasum and small and large intestines was conducted, following the procedures described in the WAAVP guidelines for evaluating the efficacy of anthelmintics in ruminants [19].

### Animals

The study animals consisted of 90 pregnant ewes, all of the Gentile di Puglia breed, between 4-6 years old and 50.0-61.9 kg body weight, with positive GIN faecal egg counts. Pregnancy diagnosis on the study animals was performed by sonography method and each ewes carried a single lamb. The selected sheep were ranked by age, numbers of previous lactations, GIN faecal egg counts, body weight, and then assigned consecutively to 3 treatment groups of 30 animals each: MOX - Group (moxidectin-treated group); IVM - Group (ivermectin-treated group); C - Group (Control - untreated group).

### Treatment Procedures

On Day 0, the MOX-group received *per os *a formulation of moxidectin 0.1%, and the IVM-group received *per os *a formulation of ivermectin, containing 0.08% w/v ivermectin. Sheep of the C-group did not receive any treatment but were subjected to the same handling procedures as the sheep of the treated groups, receiving *per os aqua fontis*. After the treatment, the study animals of the three groups were maintained together under the same conditions. In particular, they co-grazed on the same pasture throughout the study and there wasn't any change of diet during pregnancy or lactation.

### Dosage and route of administration

The animals were weighed 1 day prior to treatment (Day -1). For each treated animal, the dosage was calculated on the basis of its body weight. The anthelmintics were administered at a nominal dose rate of 0.2 mg moxidectin/kg body weight (1 ml per 5 kg) and 0.2 mg ivermectin/kg body weight (1.25 ml per 5 kg) by oral drench.

### Sampling methodology and coprological examinations

Faecal egg counts were performed on each study animal before the start of the trial (Day -3), at Days 0, 7, 14, 28, 45 after treatment and then monthly until the end of the study. Individual faecal egg counts were determined by using the Flotac technique [20] with a sensitivity of two eggs per gram (EPG) of faeces, using a sucrose flotation medium (specific gravity = 1.250).

### Coprocultures

On each sampling day, six pools of composite faecal cultures were made from five animals for each group. The animals were randomly assigned to each pool before the start of the trial. Third stage larvae were identified using the morphological keys proposed by M.A.F.F. [21]. Where a coproculture had 100 or less third stage larvae, all were identified; where a coproculture had more than 100 larvae, only 100 were identified.

### Anthelmintic efficacy

At each faecal sampling time, arithmetic mean EPG were calculated as recommended by the WAAVP guidelines for evaluating the efficacy of anthelmintics in ruminants and, for each treatment group, percent efficacy (%) was calculated in terms of Faecal Egg Count Reduction (FECR) at the different days [19,22].

### Effect of treatments on milk production

Due to the farm management practices, only the evening milk production (ml) was recorded for each study animal fortnightly for whole lactation period (April-August); it should be noted that the first two milk records were performed during the suckling period (April, 3^rd ^- 18^th^) and the other after the suckling period (May-August). Milk samples were collected into plastic containers. In order to avoid misreading by foam formation, milk samples were then slowly poured into a volumetric glass cylinder graduated to 5 ml and allowed to stand 5-10 minutes before reading. Parameters on milk quality (e.g. fat, protein) were not considered in the present study.

### Ethical approval

Ethical clearance was obtained from the Centre for Veterinary Service of the University of Naples Federico II (Ref. no. 0098377).

## Authors' contributions

GC performed the study design and revised the manuscript. VV, LM, ME, SP and VS performed the field work and the laboratory analyses. LR analyzed the results and drafted the manuscript. All authors read and approved the final manuscript.
